# Liver proteome of mice with different genetic susceptibilities to the effects of fluoride

**DOI:** 10.1590/1678-775720150364

**Published:** 2016

**Authors:** Zohaib Nisar KHAN, Aline de Lima LEITE, Senda CHARONE, Isabela Tomazini SABINO, Tatiana MARTINI, Heloísa Aparecida Barbosa da Silva PEREIRA, Rodrigo Cardoso OLIVEIRA, Marília Afonso Rabelo BUZALAF

**Affiliations:** 1 - Universidade de São Paulo, Faculdade de Odontologia de Bauru, Departamento de Ciências Biológicas, Bauru, SP, Brasil.; 2 - Universidade Federal de São Carlos, Centro de Ciências Biológicas e da Saúde, Departamento de Genética e Evolução, São Carlos, SP, Brasil.

**Keywords:** Proteomics, Fluorides, Liver, Oxidative stress

## Abstract

A/J and 129P3/J mice strains have been widely studied over the last few years because they respond quite differently to fluoride (F) exposure. 129P3/J mice are remarkably resistant to the development of dental fluorosis, despite excreting less F in urine and having higher circulating F levels. These two strains also present different characteristics regardless of F exposure.

**Objective:**

In this study, we investigated the differential pattern of protein expression in the liver of these mice to provide insights on why they have different responses to F.

**Material and Methods:**

Weanling male A/J and 129P3/J mice (n=10 from each strain) were pared and housed in metabolic cages with *ad libitum* access to low-F food and deionized water for 42 days. Liver proteome profiles were examined using nLC-MS/MS. Protein function was classified by GO biological process (Cluego v2.0.7 + Clupedia v1.0.8) and protein-protein interaction network was constructed (PSICQUIC, Cytoscape).

**Results:**

Most proteins with fold change were increased in A/J mice. The functional category with the highest percentage of altered genes was oxidation-reduction process (20%). Subnetwork analysis revealed that proteins with fold change interacted with Disks large homolog 4 and Calcium-activated potassium channel subunit alpha-1. A/J mice had an increase in proteins related to energy flux and oxidative stress.

**Conclusion:**

This could be a possible explanation for the high susceptibility of these mice to the effects of F, since the exposure also induces oxidative stress.

## INTRODUCTION

A/J and 129P3/J mice strains have been widely studied over the last few years because they respond quite differently to fluoride (F) exposure. When given the same dose of F, the A/J strain responds with a rapid onset and severe development of dental fluorosis, while the 129P3/J strain develops minimal fluorosis^8^. This was believed to be a consequence of the faster excretion of F by the 129P3/J strain. Surprisingly, a metabolic study showed that the 129P3/J mice excrete less F in urine, have higher circulating F levels and, consequently, higher bone F levels, however, they still are remarkably resistant to the development of dental fluorosis^5^.

Some differences between these strains are intrinsic to themselves and do not depend on the F exposure. For example, the A/J mice drink significantly higher volumes of water than their 129P3/J counterparts^4^, which can be explained by the increased expression of Alpha-aminoadipic semialdehyde dehydrogenase in the kidney of 129P3/J mice, regardless of F exposure. This enzyme metabolyzes irreversibly betaine aldehyde to betaine that is the most effective osmoprotectant accumulated by eukaryotic organisms to cope with osmotic stress^4^. In addition, exclusive proteins expressed in the kidney of A/J or 129P3/J mice exhibited the same profile, regardless of F exposure. This suggests that the genetic background *per se* accounts for such differences between these two strains of mice.

Liver represents the main detoxifying tissue in the body by processing, neutralizing, and eliminating toxins from the digestive tract through hepatocyte-mediated enzymatic detoxification systems. Due to these important functions, liver is one of the body’s organs most subject to injury. Thus, it is believed that the differential pattern of protein expression in the liver of A/J and 129P3/J mice can provide new insights that could explain why they respond differently when exposed to F. To achieve this, state-of-the-art shotgun proteomics combined to bioinformatics approaches were used.

## MATERIAL AND METHODS

### Animals and samples collection

Weanling male mice from the A/J and 129P3/J inbred strains (3-week-old; n=10 from each strain) were pared and housed in metabolic cages with *ad libitum* access to low-F food (AIN76A, PMI Nutrition, Richmond, IN, USA, 0.95 mg/Kg F) and deionized water for 42 days. The temperature and humidity in the climate-controlled room, which had a 12 h light/dark cycle, were 23±1°C and 40%-80%, respectively. All experimental protocols were approved by the Ethics Committee for Animal Experiments of Bauru School of Dentistry, University of São Paulo (Protocol # 031/2013). At the end of the study, the mice were anesthetized with ketamine/xylazine and livers were collected. Samples designated for proteomic analysis were stored at -80°C, while those designated for F analysis were stored at -20°C.

### Fluoride analysis in liver

Fluoride analysis was done with the ion-sensitive electrode, after hexamethyldisiloxane-facilitated diffusion^22^, exactly as previously described^20^.

### Statistical analysis

For liver F concentration, the GraphPad InStat software version 4.0 for Windows (GraphPad software Inc., La Jolla, California USA) was used. Data were analyzed by unpaired *t test* (p<0.05).

### Sample preparation for proteomic analysis

Samples were prepared for analysis as previously described^17^. The frozen tissue was homogenized in a cryogenic mill (model 6770, Spex, Metuchen, NJ, EUA). For protein extraction, liver homogenate was incubated in lysis buffer containing 7 M urea, 2 M thiourea, 4% CHAPS, 1% IPG buffer pH 3-10, 40 mM DTT for 1 h at 4°C with occasional shaking. After this period, the homogenate was centrifuged at 15,000 rpm for 30 min at 4°C and the supernatant containing soluble proteins was recovered. The proteins were precipitated using the kit *PlusOne 2D Cleanup* (GE Healthcare, Uppsala, Sweden), as recommended by the manufacturer. Pellets were resuspended in rehydration buffer (7 M urea, 2 M thiourea, 0.5% CHAPS, 0.5% IPG buffer pH 3–10, 18 mM DTT, 0.002% bromophenol blue). Twenty-five μL of liver proteins from each animal of the same group were combined to constitute a pool that was centrifuged for clarification. To each pool, 50 mM AMBIC, containing 3 M urea, were added. Each sample was filtered twice in 3 kDa AMICON (Millipore, St Charles, MO, USA). Protein quantification was measured in the pooled samples by Bradford protein assay^3^. To each sample (50 µg of total protein for each pool in a volume of 50 µL), 10 µL of 50 mM AMBIC were added. In sequence, 25 µL of 0.2% *Rapi*GEST™ (Waters Co., Manchester, UK) were added and incubated at 80°C for 15 min. Following, 2.5 µL of 100 mM DTT were added and incubated at 60°C for 30 min. Also, 2.5 µL of 300 mM IAA were added and incubated for 30 min at room temperature (under dark). Then, 10 µL of trypsin (100 ng; Trypsin Gold Mass Spectrometry, Promega, Madison, USA) were added and digestion occurred for 14 h at 37°C. After digestion, 10 µl of 5 % TFA were added, incubated for 90 min at 37°C and the sample was centrifuged (14,000 rpm for 30 min). The supernatant was collected and 5 µL of ADH (1 pmol/µL) plus 85 µL 3% ACN were added.

### LC-MS/MS and bioinformatics analyses

Separation and identification of peptides were performed on a nanoAcquity UPLC-Xevo QTof MS system (Waters, Manchester, UK), exactly as previously described^15^. Difference in expression among the groups was obtained using PLGS software and expressed as p<0.05 for down-regulated proteins 1-p>0.95 for up-regulated proteins (Table 1). Bioinformatics analysis was performed, as reported earlier^1^ ^,^ ^15^ ^,^ ^17^ ^-^ ^19^. Briefly, Uniprot protein ID accession numbers were mapped back to their associated encoding Uniprot gene entries for the comparison A/J X 129P3/J. Gene Ontology annotation of Broad Biological Process was performed using Cluego v2.0.7 + Clupedia v1.0.8, a Cytoscape plugin. Uniprot IDs were uploaded to Table 1 and analyzed with default parameters, which specify a Enrichment (right-sided hypergeometric test) correction method using Bonferroni step down, analysis mode “Function” and load gene cluster list for *Mus musculus*, Evidence Codes “All”, set networking specificity “medium” (GO levels 3 to 8) and KappaScoreThreshold 0.03. The protein-protein interaction network was downloaded from PSICQUIC, built in Cytoscape version 3.0.2 and constructed as proposed by Millan^18^ (2013). A network was then created, providing global view of potentially relevant interacting partners of proteins whose abundances change.

**Table 1:** Identified proteins with expression significantly altered in the liver of mice of group A/J control vs. 129 control (0 ppm F)

			Foldchange		
^a^Access	Gene	Protein name description	PLGS score	A/J 0 ppm	129P3/ J 0 ppm
Number	name				
Q921H8	Acaa1a	3-ketoacyl-CoA thiolase A, peroxisomal	195.3	1.65	-1.65
Q8VCH0	Acaa1b	3-ketoacyl-CoA thiolase B, peroxisomal	195.3	1.70	-1.70
Q8BWT1	Acaa2	3-ketoacyl-CoA thiolase, mitochondrial	189.2	1,42	-1,42
P63038	Hspd1	60 kDa heat shock protein, mitochondrial	153.6	1.55	-1.55
P20029	Hspa5	78 kDa glucose-regulatedprotein	254.4	1.43	-1.43
P68033	Actc1	Actin, alpha cardiacmuscle 1	630.1	1.28	-1.28
P68134	Acta1	Actin, alpha skeletalmuscle	630.1	1.28	-1.28
P62737	Acta2	Actin, aorticsmoothmuscle	60.2	1.35	-1.35
P60710	Actb	Actin, cytoplasmic 1	62.4	1.25	-1.25
P63260	Actg1	Actin, cytoplasmic 2	62.4	1.26	-1.26
P63268	Actg2	Actin, gamma-enteric smooth muscle	60.2	1.34	-1.34
P47738	Aldh2	Aldehydedehydrogenase, mitochondrial	72.6	1.67	-1.67
P17182	Eno1	Alpha-enolase OS=Mus musculus	129.4	1.46	-1.46
P16460	Ass1	Argininosuccinatesynthase	58.6	1.28	-1.28
P05202	Got2	Aspartateaminotransferase, mitochondrial	79.3	1.34	-1.34
Q03265	Atp5a1	ATP synthase subunit alpha, mitochondrial	74.7	1.43	-1.43
P56480	Atp5b	ATP synthasesubunit beta, mitochondrial	138.6	1.35	-1.35
O35490	Bhmt	Betaine--homocysteine S-methyltransferase 1	40.6	1.23	-1.23
Q8C196	Cps1	Carbamoyl-phosphate synthase [ammonia], mitochondrial	269.2	1.39	-1.39
Q63880	Ces3a	Carboxylesterase 3A	336.9	1.46	-1.46
Q8VCU1	Ces3b	Carboxylesterase 3B	139.1	1.65	-1.65
P24270	Cat	Catalase	260.8	1.62	-1.62
Q8R0Y6	Aldh1l1	Cytosolic 10-formyltetrahydrofolate dehydrogenase	53.1	1.55	-1.55
Q9DCW4	Etfb	Electron transfer flavoprotein subunit beta	174.4	1.48	-1.48
P10126	Eef1a1	Elongationfactor 1-alpha 1	245.5	1.39	-1.39
P70694	Akr1c6	Estradiol 17 beta-dehydrogenase 5	207.5	1.48	-1.48
Q91XD4	Ftcd	Formimidoyltransferase-cyclodeaminase	121.1	3.82	-3.82
Q91Y97	Aldob	Fructose-bisphosphatealdolase B	96.1	1.62	-1.62
P35505	Fah	Fumarylacetoacetase	136.0	1.46	-1.46
P26443	Glud1	Glutamatedehydrogenase 1, mitochondrial	467.9	1.84	-1.84
P10649	Gstm1	Glutathione S-transferase Mu 1	129.1	1.26	-1.26
P15626	Gstm2	Glutathione S-transferase Mu 2	109.8	1.32	-1.32
P48774	Gstm5	Glutathione S-transferase Mu 5	109.8	1.32	-1.32
P19157	Gstp1	Glutathione S-transferase P 1	317.2	-0.66	0.66
P63017	Hspa8	Heat shock cognate 71 kDa protein	275.2	1.36	-1.36
P01942	Hba	Hemoglobinsubunit alpha	1252.1	-0.85	0.85
P02104	Hbb-y	Hemoglobinsubunit epsilon-Y2	854.2	-0.48	0.48
Q8CGP6	Hist1h2ah	Histone H2A type 1-H	193.0	1.22	-1.22
Q64522	Hist2h2ab	Histone H2A type 2-B	241.3	1.51	-1.51
P62806	Hist1h4a	Histone H4	88.1	1.54	-1.54
P54869	Hmgcs2	Hydroxymethylglutaryl-CoAsynthase, mitochondrial	292.1	1.22	-1.22
P11588	Mup1	Major urinaryprotein 1	815.0	-0.53	0.53
B5X0G2	Mup17	Major urinaryprotein 17	824.6	-0.54	0.54
P11589	Mup2	Major urinaryprotein 2	815.0	-0.54	0.54
P11591	Mup5	Major urinaryprotein 5	389.7	-0.57	0.57
P02762	Mup6	Major urinaryprotein 6	815.0	-0.53	0.53
P04938	Mup8	Major urinary proteins 11 and 8 (Fragment)	815.0	-0.54	0.54
P08249	Mdh2	Malatedehydrogenase, mitochondrial	247.9	1.45	-1.45
Q64374	Rgn	Regucalcin	107.2	1.36	-1.36
P24549	Aldh1a1	Retinaldehydrogenase 1	208.9	1.49	-1.49
P07724	Alb	Serumalbumin	108.5	1.34	-1.34
P00329	Adh1	Alcoholdehydrogenase 1	163.3	+	-
Q61234	Snta1	Alpha-1-syntrophin	77.6	+	-
Q8VCT3	Rnpep	Aminopeptidase B	73.8	+	-
Q9D3D9	Atp5d	ATP synthasesubunit delta, mitochondrial	183.6	+	-
Q62210	Birc2	Baculoviral IAP repeat-containing protein 2	65.9	+	-
Bad	Q61337	Bcl2 antagonist of cell death	116.2	-	+
P21550	Eno3	Beta-enolase	161.0	+	-
P34914	Ephx2	Bifunctionalepoxidehydrolase 2	441.9	+	-
Q8R1G2	Cmbl	Carboxymethylenebutenolidasehomolog	73.2	+	-
Q61686	Cbx5	Chromoboxproteinhomolog 5	96.9	+	-
Q3V079	Ccdc176	Coiled-coil domain-containing protein 176	66.5	+	-
P50172	Hsd11b1	Corticosteroid 11-beta-dehydrogenase isozyme 1	100.4	+	-
Cth	Q8VCN5	Cystathioninegamma-lyase	100.5	-	+
P48771	Cox7a2	Cytochrome c oxidase subunit 7A2, mitochondrial	185.6	+	-
P10518	Alad	Delta-aminolevulinicaciddehydratase	316.8	+	-
Q9DBT9	Dmgdh	Dimethylglycinedehydrogenase, mitochondrial	89.4	+	-
Q99LC5	Etfa	Electron transfer flavoprotein subunit alpha, mitochondrial	77.6	+	-
Q9ER73	Elp4	Elongatorcomplexprotein 4	103.4	+	-
P63242	Eif5a	Eukaryotic translation initiation factor 5A-1	104.8	+	-
Q9QXD6	Fbp1	Fructose-1,6-bisphosphatase 1	154.4	+	-
P17183	Eno2	Gamma-enolase	159.3	+	-
Q3UHD2	Gfod1	Glucose-fructose oxidoreductase domain-containing protein 1	83.6	+	-
P11352	Gpx1	Glutathioneperoxidase 1	419.0	+	-
P24472	Gsta4	Glutathione S-transferase A4	127.0	+	-
Q9QYE6	Golga5	Golginsubfamily A member 5	103.4	+	-
P07901	Hsp90aa1	Heat shock protein HSP 90-alpha	67.4	+	-
P11499	Hsp90ab1	Heat shock protein HSP 90-beta	107.9	+	-
P68433	Hist1h3a	Histone H3.1	163.6	+	-
P84228	Hist1h3b	Histone H3.2	163.6	+	-
P84244	H3f3a	Histone H3.3	163.6	+	-
P02301	H3f3c	Histone H3.3C	163.6	+	-
Hgd	O09173	Homogentisate 1,2-dioxygenase	95.6	-	+
Hadh	Q61425	Hydroxyacyl-coenzyme A dehydrogenase, mitochondrial	183.9	-	+
Q5U5V2	Hykk	Hydroxylysinekinase	78.0	+	-
Q8BLR9	Hif1an	Hypoxia-induciblefactor 1-alpha inhibitor	96.3	+	-
O88844	Idh1	Isocitratedehydrogenase [NADP] cytoplasmic	69.5	+	-
Q9CPU0	Glo1	Lactoylglutathionelyase	203.5	+	-
P06151	Ldha	L-lactatedehydrogenase A chain	153.0	+	-
Acsl1	P41216	Long-chain-fatty-acid--CoA ligase 1	48.0	-	+
Q9DB40	Med27	Mediator of RNA polymerase II transcription subunit 27	68.9	+	-
Q8BPT6	Immp2l	Mitochondrial inner membrane protease subunit 2	65.7	+	-
Myef2	Q8C854	Myelinexpressionfactor 2	44.9	-	+
Q9DC69	Ndufa9	NADH dehydrogenase [ubiquinone] 1 alpha subcomplexsubunit 9, mitochondrial	79.2	+	-
Ncoa5	Q91W39	Nuclear receptor coactivator 5	67.7	-	+
P11725	Otc	Ornithinecarbamoyltransferase, mitochondrial	217.0	+	-
O08807	Prdx4	Peroxiredoxin-4	391.3	+	-
Prdx5	P99029	Peroxiredoxin-5, mitochondrial	174.7	-	+
O08709	Prdx6	Peroxiredoxin-6	321.1	+	-
P09411	Pgk1	Phosphoglyceratekinase 1	106.8	+	-
Pgap2	Q3TQR0	Post-GPI attachment to proteins factor 2	60.0	-	+
Prdm12	A2AJ77	PR domainzincfingerprotein 12	43.7	-	+
Q80U40	Rimbp2	RIMS-bindingprotein 2	74.3	+	-
B2RY56	Rbm25	RNA-bindingprotein 25	80.8	+	-
Q91X83	Mat1a	S-adenosylmethionine synthase isoform type-1	177.4	+	-
Q99J08	Sec14l2	SEC14-like protein 2	106.4	+	-
P47758	Srprb	Signal recognition particle receptor subunit beta	68.7	+	-
Hspa9	P38647	Stress-70 protein, mitochondrial	119.8	-	+
Q8K2B3	Sdha	Succinatedehydrogenase [ubiquinone] flavoproteinsubunit, mitochondrial	74.3	+	-
Q62264	Thrsp	Thyroid hormone-inducible hepatic protein	180.0	+	-
P97360	Etv6	Transcriptionfactor ETV6	64.7	+	-
Tmem42	Q9CR22	Transmembraneprotein 42	110.6	-	+
Tpi1	P17751	Triosephosphateisomerase	149.7	-	+
Q9D6F9	Tubb4a	Tubulin beta-4A chain	101.3	+	-
P68372	Tubb4b	Tubulin beta-4B chain	109.0	+	-
Ube2w	Q8VDW4	Ubiquitin-conjugatingenzyme E2 W	102.0	-	+
Q5QNV8	Heatr9	Uncharacterizedprotein C17orf66 homolog	91.1	+	-
N/A	Q8C4X7	UPF0258 protein KIAA1024-like homolog	38.4	-	+
P25688	Uox	Uricase	92.7	+	-

The identified proteins are organized according to alphabetical order. Relative differential is indicated by + sign, when the protein is up-regulated and by - sign, when the protein is down-regulated in the respective comparison. ^a^Identification is based on protein ID from UniProt protein database (http://www.uniprot.org/)

## RESULTS

### Liver F analysis

Mean±SD liver F concentrations found in 129P3/J mice (0.022±0.003 µg/g) were significantly higher than those found in A/J mice (0.015±0.002 µg/g) (*t*=4.929, p=0.0006).

### Liver proteome profile and identification of differentially expressed proteins

Table 1 shows proteins with expression changes in A/J and 129P3/J mice. In general, most proteins with fold change were increased in A/J mice.

### Gene ontology annotation

**Figure 1:**
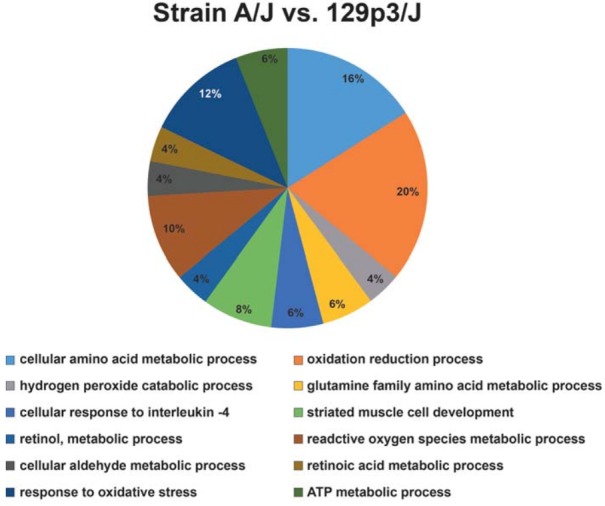
Functional distribution of proteins identified with differential expression in liver of mice belonging to A/J vs. 129p3/J strains. Categories of proteins based on GO annotation Biological Process. Terms significant (Kappa=0.03) and distribution according to percentage of number of genes association

Figure 1 shows the functional classification according to the biological process with the most significant term. Twelve categories were observed. Among them, the category with the highest percentage of genes was oxidation-reduction process (20%), followed by cellular amino acid metabolic process (16%) and response to oxidative stress (12%).

### Protein-protein interaction network

**Figure 2:**
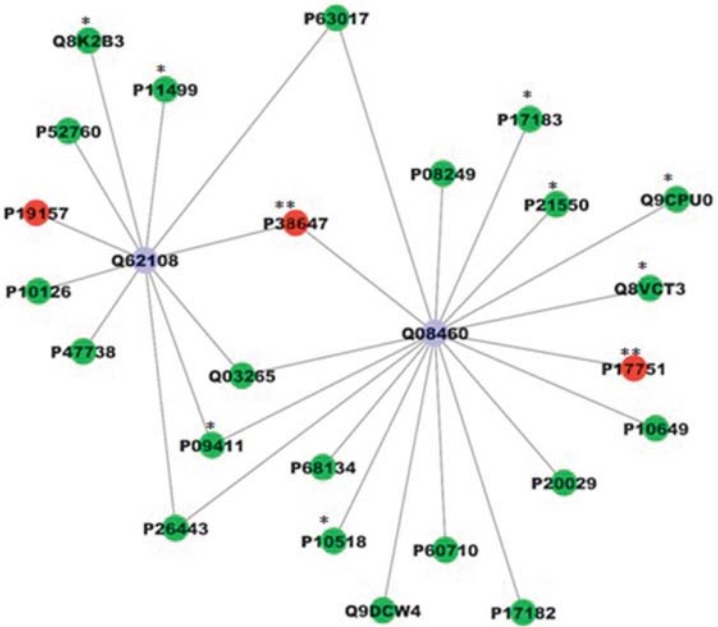
Subnetworks generated by VizMapper for each comparison – A Group A/J vs. 129p3/J. Color of node and * indicate the differential expression of the respective protein, for each comparison. Red and green nodes indicate protein down-regulation and up-regulation, respectively, while * and ** indicate presence and absence of protein, respectively, in the respective group. Purple node indicates proteins presenting interaction but that were not identified in the present study. The access numbers in nodes correspond to: P68134- (Acta1)Actin, alpha skeletal muscle; P10518- (Alad) Delta-aminolevulinic acid dehydratase; Q9DCW4- (Etfb) Electron transfer flavoprotein subunit beta; P60710- (Actb) Actin, cytoplasmic 1; P17182- (Eno1) Alpha-enolase; P20029- (Hspa5) 78 kDa glucose-regulated protein; P10649- (Gstm1) Glutathione S-transferase Mu 1; P17751- (Tpi1) Triosephosphate isomerase; Q8VCT3- (Rnpep) Aminopeptidase B; Q9CPU0- (Glo1) Lactoylglutathionelyase; P21550- (Eno3) Beta-enolase; P17183- (Eno2) Gamma-enolase; P08249- (Mdh2) Malate dehydrogenase; P63017- (Hspa8) Heat shock cognate; P38647- (Hspa9) Stress-70 protein; Q03265- (Atp5a1) ATP synthase subunit alpha; P09411- (Pgk1) Phosphoglycerate kinase 1; P26443- (Glud1) Glutamate dehydrogenase 1; P47738- (Aldh2) Aldehyde dehydrogenase; P10126- (Eef1a1) Elongation factor 1-alpha 1; P19157- (Gstp1) Glutathione S-transferase P 1; P52760- (Hrsp12) Ribonuclease; Q8K2B3- (Sdha) Succinate dehydrogenase; P11499- (Hsp90ab1) Heat shock protein; Q62108- (Dlg4) Disks large homolog 4; Q08460- (Kcnma1) Calcium-activated potassium channel subunit alpha-1

For the comparison displayed above, a network was created; employing all the interactions found in the search conducted using PSICQUIC. After the global network was created, nodes and edges were filtered using the specification for *Mus musculus* taxonomy (10090). The value of fold change and also the p-value were added in new columns. The ActiveModules 1.8 plug-in to Cytoscape was used to make active modules connected subnetworks within the molecular interaction network whose genes presented significant coordinated changes in fold changes and p-value, as shown in the original proteomic analysis. Figure 2 shows the subnetwork generated by VizMapper. As can be seen, most proteins with fold change present interaction with Disks large homolog 4 (Q62108; 11 proteins) and Calcium-activated potassium channel subunit alpha-1 (Q08460; 18 proteins).

## DISCUSSION

129P3/J mice interestingly have been reported to excrete less F and as consequence to have higher circulating F levels, bone and enamel F levels and they still are remarkably resistant to the development of dental bfluorosis^5^ ^,^ ^7^ ^-^ ^8^ ^,^ ^12^. In this study, even without administration of F through the drinking water and with consumption of a low-F diet, 129P3/J mice had significantly higher liver F concentrations, which might have been due to the residual amounts of F present in their diets and is in-line with the metabolic characteristics of this strain regarding F^4^ ^-^ ^5^.

In this study, proteomic analysis of liver of 129P3/J and A/J mice was employed to provide insights into the possible mechanisms that could explain the differential metabolic handling and effects of F in these two strains. It has been shown that even without exposure to F, A/J mice present a higher retention of proteins in the maturing enamel^9^. For this reason, the mice were not treated with F, because we wanted to see differences in the liver proteome profile that were intrinsic to the strains. Most proteins with fold change were increased in the A/J mice (Table 1), with fold changes ranging between 1 and 2. *Formimidoyltransferase-cyclodeaminase*, however, was increased 3.82 times in A/J mice. This enzyme is a liver-specific antigen recognized by sera of patients with autoimmune hepatitis^14^ and is found down-regulated in hepatocellular carcinoma^16^. *Formimidoyltransferase-cyclodeaminase* has two enzymatic functions. In one of them, formiminotetrahydrofolate and glutamate are produced. Through its cyclodeaminase function, the enzyme breaks down formiminotetrahydrofolate, involved in the synthesis of purines and pyrimidines, and amino acids (UNIPROT). Thus, the increase in this enzyme might explain the increased expression of other liver proteins in A/J mice due to higher supply of nucleotides and amino acids.

Remarkably, the functional category with the highest percentage of altered genes was oxidation-reduction process. The increase of proteins such as ATP synthase subunit alpha, mitochondrial, Heat shock cognate 71 kDa protein, Electron transfer flavoprotein subunit beta, Alpha-enolase, Beta-enolase, Gamma-enolase and, Malate dehydrogenase in the A/J mice indicate an increased energy flux in this strain, which might generate oxidative stress. This can be confirmed by the concomitant increase in GRP78, which suggests endoplasmic reticulum (ER) stress^20^. ER stress occurs when nascent proteins are misfolded or not folded properly, leading to the initiation of the unfolded protein response, as the unfolded proteins accumulate in the ER^13^. It has been demonstrated that F is able to induce an ER stress response in the LS8 ameloblast-derived cell line, which could be implicated in the pathogenesis of dental fluorosis^13^. In addition, administration of F through the drinking water is able to increase the expression of GRP78 in the liver of rats^20^. Thus, considering that A/J mice present an increased energy flux and tendency to oxidative stress even without exposure to F, this exposure has been shown to worsen oxidative stress^20^, which can implicate in the pathogenesis of dental fluorosis^8^, this can be a hypothesis for the high susceptibility of the A/J to the effects of F.

The proteins in the center of the protein-protein interaction network are related to potassium channels. One of them (calcium-activated potassium channel subunit alpha-1) is a potassium channel activated either by membrane depolarization or increase in cytosolic Ca^2+^ that mediates export of K^+^. It is also activated by the concentration of cytosolic Mg^2+^. Its activation dampens the excitatory events that elevate the cytosolic Ca^2+^ concentration and/or depolarize the cell membrane. Therefore, it contributes to the repolarization of the membrane potential and plays a key role in controlling excitability in a number of systems, such as regulation of the contraction of smooth muscle^21^, the tuning of hair cells in the cochlea^6^, regulation of transmitter release^6^ and innate immunity^2^. The other one is Disks large homolog 4 that is required for synaptic plasticity associated with NMDA (N-methyl-D-aspartate) receptor signaling^11^. It interacts with shaker-type potassium channels and the cytoplasmic tail of NMDA receptor subunits. At first glance, it may seem odd the presence of a protein associated with the nervous system in the center of the network in this study. However, we must consider that liver failure leaves to the accumulation of ammonia, which affects the cerebral function^10^. As mentioned above, A/J mice presented several proteins related to the energy flux increased in the liver, which might have caused oxidative stress and contributed to liver damage, which in turn might have provoked cerebral alterations. Since this was a preliminary exploratory work, future studies comparing the proteomic profile of the brain of these mice strains should be conducted to add new light into this topic. Also, additional studies should be done to quantify, by other techniques, the proteins with changing expression in this study. Despite being an exploratory study, the lack of additional techniques to confirm the proteins with altered expression identified by nLC-MS/MS might be considered a limitation of this study.

## CONCLUSIONS

In conclusion, A/J mice had an increase in proteins related to energy flux and oxidative stress. This could be a possible explanation for the high susceptibility of these mice to the effects of F, since F exposure also induces oxidative stress.

## References

[1] Bauer-Mehren A (2013). Integration of genomic information with biological networks using Cytoscape. Methods Mol Biol.

[2] Butler A, Tsunoda S, McCobb DP, Wei A, Salkoff L (1993). mSlo, a complex mouse gene encoding "maxi" calcium-activated potassium channels. Science.

[3] Bradford MM (1976). A rapid and sensitive method for the quantitation of microgram quantities of protein utilizing the principle of protein-dye binding. Anal Biochem.

[4] Carvalho JG, Leite AL, Peres-Buzalaf C, Salvato F, Labate CA, Everett ET (2013). Renal proteome in mice with different susceptibilities to fluorosis. PLoS One.

[5] Carvalho JG, Leite AL, Yan D, Everett ET, Whitford GM, Buzalaf MA (2009). Influence of genetic background on fluoride metabolism in mice. J Dent Res.

[6] Cabo R, Zichichi R, Viña E, Guerrera MC, Vázquez G, García-Suárez O (2013). Calcium-activated potassium channel SK1 is widely expressed in the peripheral nervous system and sensory organs of adult zebrafish. Neurosci Lett.

[7] Charone S, Leite AL, Peres-Buzalaf C, Fernandes MS, Almeida LF, Graeff MS (2016). Proteomics of secretory and maturation stage enamel of genetically distinct mice. Caries Res.

[8] Everett ET, McHenry MA, Reynolds N, Eggertsson H, Sullivan J, Kantmann C (2002). Dental fluorosis: variability among different inbred mouse strains. J Dent Res.

[9] Everett ET, Yan D, Weaver M, Liu L, Foroud T, Martinez-Mier EA (2009). Detection of dental fluorosis-associated quantitative trait Loci on mouse chromosomes 2 and 11. Cells Tissues Organs.

[10] Felipo V (2013). Hepatic encephalopathy: effects of liver failure on brain function. Nat Rev Neurosci.

[11] Halff AW, Gómez-Varela D, John D, Berg DK (2014). A novel mechanism for nicotinic potentiation of glutamatergic synapses. J Neurosci.

[12] Kobayashi CA, Leite AL, Peres-Buzalaf C, Carvalho JG, Whitford GM, Everett ET (2014). Bone response to fluoride exposure is influenced by genetics. PLoS One.

[13] Kubota K, Lee DH, Tsuchiya M, Young CS, Everett ET, Martinez-Mier EA (2005). Fluoride induces endoplasmic reticulum stress in ameloblasts responsible for dental enamel formation. J Biol Chem.

[14] Lapierre P, Hajoui O, Homberg JC, Alvarez F (1999). Formiminotransferase cyclodeaminase is an organ-specific autoantigen recognized by sera of patients with autoimmune hepatitis. Gastroenterology.

[15] Leite AL, Lobo GV, Pereira HA, Fernandes MS, Martini T, Zucki F (2014). Proteomic analysis of gastrocnemius muscle in rats with streptozotocin-induced diabetes and chronically exposed to fluoride. PLoS One.

[16] Liang CR, Leow CK, Neo JC, Tan GS, Lo SL, Lim JW (2005). Proteome analysis of human hepatocellular carcinoma tissues by two-dimensional difference gel electrophoresis and mass spectrometry. Proteomics.

[17] Lobo JG, Leite AL, Pereira HA, Fernandes MS, Peres-Buzalaf C, Sumida DH (2015). Low-level fluoride exposure increases insulin sensitivity in experimental diabetes. J Dent Res.

[18] Millan PP (2013). Visualization and analysis of biological networks. Methods Mol Biol.

[19] Orchard S (2012). Molecular interaction databases. Proteomics.

[20] Pereira HA, Leite AL, Charone S, Lobo JG, Cestari TM, Peres-Buzalaf C (2013). Proteomic analysis of liver in rats chronically exposed to fluoride. PLoS One.

[21] Sánchez-Pastor E, Andrade F, Sánchez-Pastor JM, Elizalde A, Huerta M, Virgen-Ortiz A (2014). Cannabinoid receptor type 1 activation by arachidonylcyclopropylamide in rat aortic rings causes vasorelaxation involving calcium-activated potassium channel subunit alpha-1 and calcium channel, voltage-dependent, L type, alpha 1C subunit. Eur J Pharmacol.

[22] Taves DR (1968). Separation of fluoride by rapid diffusion using hexamethyldisiloxane. Talanta.

